# Perception and practice of breastfeeding in public in an urban community in Accra, Ghana

**DOI:** 10.1186/s13006-018-0161-1

**Published:** 2018-05-18

**Authors:** Justine Boatemaa Coomson, Richmond Aryeetey

**Affiliations:** School of Public Health, University of Ghana, P.O. Box LG 13, Legon, Accra Ghana

**Keywords:** Breastfeeding in public, Urban, Perception, Experience, Stigma, Women, Ghana

## Abstract

**Background:**

Reported stigmatization and confrontation (verbal and aggressive), of women when breastfeeding in public spaces constitutes a barrier to the recommendation to breastfeed infants on demand. While such stigma of breastfeeding in public has been reported more commonly in Western country media, there is no documented evidence of this stigma in developing country settings. The current study describes community perception and experience of breastfeeding in public in Accra, Ghana. A secondary objective is to explore factors associated with breastfeeding in public.

**Methods:**

A mixed methods design comprising a survey (*n* = 300), five Focus Group Discussions (FGD) with lactating women (*n* = 50), and nine In-Depth Interviews (IDI) with adult males (*n* = 5) and female health workers (*n* = 4) were also conducted. All data were collected between May and June, 2016 in the Ayawaso-West Sub-Metropolis, Accra. Data on sociodemographic characteristics; obstetric and breastfeeding history; and also perception, experience, and practice of breastfeeding in public, were collected. FGDs and IDI data were triangulated with survey data and presented using descriptive and analytical statistics and content analysis.

**Results:**

Majority of the survey respondents (92%) reported ever practicing breastfeeding in public. However, some reported feeling uncomfortable (52%), embarrassed (27%), or stigmatized (15%). Nevertheless, 72% of respondents felt they should be able to breastfeed anywhere. Almost all respondents (81%) reported covering their breasts when breastfeeding in public, and 70% felt private places should be used when they breastfed in public. Women in FGDs and IDI mentioned that they bottle feed their children in public places as a way to overcome the challenges of breastfeeding in public. Otherwise, they breastfeed in public because their children need to feed. Women were more likely to breastfeed in public if they reported support from a spouse (OR 3.99, 95% CI 1.50, 10.57) or other family members (OR 3.27, 95% CI 1.31, 8.18).

**Conclusions:**

Although women reported discomfort associated with breastfeeding in public, the practice is common. Awareness creation is needed to empower women to comfortably breastfeed and to sustain societal support of breastfeeding in public.

## Background

The World Health Organization recommends exclusive breastfeeding for the first six months of life, and thereafter, continued breastfeeding plus appropriate complementary feeding until age two or beyond [1]. Optimal breastfeeding practice, including early initiation and exclusive breastfeeding, is associated with positive health effects on both mother and child [1–8]. One way to achieve optimal breastfeeding is to promote ‘breastfeeding on demand’. Breastfeeding on demand is promoted to ensure infants obtain adequate intake of breast milk [9].

Media reports in some Western countries have shown that women who breastfeed in public places experience stigma in the form of verbal, and even physical abuse [10, 11]. ‘Public places’ refers to any location outside a private home (including restaurants, workplaces, shopping centers and public transport, etc.) or within a home, but which is visually accessible to other community members. Stigmatizing women who BIP can serve as a barrier to recommended breastfeeding practices [12, 13].

Almost all children in Ghana are breastfed. Exclusive breastfeeding and early initiation rates are however low. The 2014 Demographic and Health Survey estimates that only 52% of infants are exclusively breastfed and 56% are put to the breast with one hour of birth [14]. As more women work outside the home, mothers may be separated from young infants for extended periods and may take their children along with them to work. This necessitates breastfeeding their young children outside the home. However, there is currently no evidence on perceptions and practice of breastfeeding in public (BIP) in a developing country setting like Ghana. There is also no evidence on how breastfeeding occurs in other settings outside the home (markets, worship centres, etc) as well as its implications for breastfeeding practice. The current study assessed perceptions and practice of BIP as well as the drivers of breastfeeding in public.

## Methods

A descriptive cross-sectional study design using mixed methods including Focus Group Discussions (FGD), key informant interviews (KII) and Indepth Interviews (IDI) and a survey was used to explore the perceptions, experiences, and practices of women and men, 18 years and older, concerning breastfeeding in public. This study was undertaken in the Ayawaso-West Sub-Metropolitan area in the Greater Accra Region of Ghana. The Sub-Metropolitan Area has an estimated population of 70,667 of which 24,917 are adult women. The Sub-Metropolitan area has four community-based child welfare clinic (CWC) sites at Shiashie, Mempeasem, Bawaleshie, and Legon. These CWCs operate once in a week at each location, and provide growth monitoring and promotion, vaccination, and health and nutrition education services.

Survey respondents were consecutively selected from all four CWCs, and also from households in the community. Women between ages 15–49 years, who had a live birth in the last five years, and had breastfed that child for at least three months, and who had given consent to participate were enrolled.

A total of 300 women completed a semi structured questionnaire that was used to collect information on socio-demographic and obstetric characteristics, as well as perceptions, experiences, and practice of breastfeeding in public. Questions on perception and behaviors relating to BIP were adapted from the Centers for Disease Control’s Breastfeeding Health Styles Survey, 2010 [15]. Data collection for the survey occured between May and June, 2016. All FGD and IDI were conducted in June, 2016.

In addition, fifty women participated in five FGDs made up of ten women each. Five IDI's with purposively selected adult males (visitors to Legon Hospital) and four KII's with nurses and midwives in the CWC of the University hospital at Legon, were conducted to obtain information regarding their perceptions of breastfeeding in public.

FGD's were facilitated by a trained interviewer and assisted by a note taker. The facilitator led the groups to discuss their perceptions and experiences of BIP and its relation to exclusive breastfeeding. Each FGD lasted 90 min and IDI's and KII's lasted, averagely, 40 min. All the FGD's, KII's and IDI's were conducted using an interview guide designed for the study. Interviews were tape recorded and detailed notes were also taken.

Completed survey questionnaires were cross checked by the lead researcher for completeness before the respondents left the site. Quantitative data obtained from the survey were entered into excel. The entered data was cross checked with entries in the completed questionnaires for consistency before analysis was done. Stata version13 (Startacorp, USA) was used for statistical analyses. Descriptive statistics such as means and percentages were used to summarize and present demographic data of the respondents. Logistic regression models including socio-demographic and obstetric characteristics of the respondents were entered as explanatory variables to identify factors associated with BIP perception and practice. Those parameters that were significantly associated with perception or practice of BIP (at *p* < 0.05) were selected and included in the respective multiple logistic regression model.

Data obtained from both the FGD's and IDI's were analyzed manually. The audio records were translated and transcribed into English. The transcripts, with the hand written notes taken during the interviews, were manually read by two independent investigators, coded, cross-referenced and compared across the different IDI's and FGD's. Unique numbering was used to code the themes. The frequency of the coded text was considered as an indication of thematic unit significance. Their individual codes were checked with each other for consistency and to arrive at consensus on the emergent themes. Perceptions and experiences of the respondents emerging from the coded data were pooled together as the perception of women and men regarding breastfeeding in public.

Women’s responses to Likert-style questions on BIP perception in the survey were scored on a scale, and a composite score was obtained from a sum of all perception questions. Women’s perception was categorized as positive (if composite score on perception was at least 50% of the total score) or negative (if the score was less than 50% of the total score). Similar scoring categories were computed for family, spouse, or close friends’ support for BIP, and was based on women’s responses to questions which assessed their family’s, or friend’s attitude towards BIP. A score of at least 50% of the total was considered as strong family support, and a score less than 50% was considered as poor.

## Results

### Demographic and obstetric characteristics of survey participants

This study included 300 women in the survey, and 53 women and 5 men in qualitative interviews; thier ages ranged between 18 and 49 years. The majority of the women (63%) in the survey were between 25 and 34 years and 94.0% were in spousal relationships. Sixty-four percent had completed secondary education or higher and 83% were employed; 50.3% were self-employed (Table 1). Thirty-six percent of them were first-time mothers and 50.3% had two or three children at the time of the study.

**Table 1:** Socio-demographic and obstetric characteristics of study participants

Characteristics of women	*N*	Percentage
Age categories		
< 25	28	9.3
25–29	92	30.7
30–34	97	32.3
35–39	64	21.3
≥ 40	19	6.3
Education		
No education	15	5.0
Elementary	122	40.7
Secondary+	163	54.3
Occupation		
Unknown occupation	42	14.0
Skilled manual	79	26.3
Professional/technical/managerial	69	23.0
Clerical/sales and services	99	33.0
Unskilled manual	11	3.7
Type of Employment		
No employment	51	17.0
Self employed	151	50.3
Employed by other	98	32.7
Marital status		
Not in marital relationship	18	6.0
In marital relationship	282	94.0
Number of live births		
1 child	109	36.3
2–3 livebirths	151	50.3
More than 3	40	13.3
Antenatal clinic attendance		
< 4 visits	5	1.7
≥ 4 visits	295	98.3
Median duration of breastfeeding last/index child		12 (IQR = 8–18)
Preparation for breastfeeding		
Not prepared to breastfeed	8	2.7
Somewhat prepared to breastfeed	24	8.0
Well prepared to breastfeed	268	89.3
Informed about breastfeeding during antenatal care		
Not informed	16	5.3
Well informed	286	94.7
Informed about breastfeeding in public during antenatal care		
Not informed	144	48.0
Somewhat informed	67	22.3
Well informed	89	29.7

The median duration of breastfeeding of the index child of survey participants was 12 months; IQR=8-18. The majority (98.3%) of the mothers attended antenatal clinic (ANC) at least four times prior to delivery. Although majority (94.7%) of survey respondents reported they had been well informed about breastfeeding at the ANC, 10.7% felt they were unprepared to breastfeed their last child, at the time of delivery. About a third (29.7%) recalled they had received counseling about breastfeeding in public at the ANC (Table 1). The majority (70%) of women in the survey had family or close friends also breastfeeding at the time they were breastfeeding. Also, 57.7% of women had family or close friends who supported exclusive breastfeeding for six months. Three of the men who were included in the in-depth interviews were married and had at least one child each. The four health workers interviewed, had all worked in the health facility for at least one year. Three of the health worker respondents were female, and two were mothers.

### Women’s perception of breastfeeding in public

Public places were described in the in-depth interviews as any venue which is physically outside the home or a home with non-household members around.

*Public can be your sitting room when you have visitors who are not usual members of your home. It can also be outside your home, such as your workplace, in public transport, church, etc.* ***Male in-depth interview respondent, University Hospital, Legon.***

The majority of the women in the survey (72%) felt they should be able to breastfeed anywhere their child needed to feed. The FGDs also revealed that while some women had no difficulty with BIP, others were uncomfortable doing this. Some of these women in the FGD’s reported that BIP was acceptable only if they know how to discreetly breastfeed without making others or themselves uncomfortable. Some women in the KII and FGDs described their perception of breastfeeding in public.

*I feel it is acceptable for me to breastfeed in public because I know how to be discreet about it. You won’t even see the breast.* ***Female FGD respondent, University Hospital, Legon***.

*It is something that is uncomfortable to do, it’s the same feeling everywhere because it’s not so easy exposing your breast in public for all others to see, especially, people you do not know.* ***Female KII respondent, University hospital, Legon.***

Women who found it difficult to breastfeed in public reported feeling shy or uncomfortable with exposing their breast in public places.

*The breast itself is a private part so shyness is the main issue with BIP, because you feel shy to expose your private parts in public.* ***Female FGD respondent, University Hospital, Legon.***

Others mentioned perceived problems with the breast as a reason why women avoid breastfeeding in public:

*With me, my breast does not look nice. So I don’t like to bring my breast out anyhow. I know that my breast is not nice that is why I will not breastfeed in public.* ***Female FGD respondent, Mempeasem.***

About 13% of the women in the survey indicated it was not acceptable to breastfeed in public and 31% reported they were uncomfortable when they had watched other women breastfeed in public.

While some male respondents in the interviews indicated feeling discomfort when a woman is breastfeeding, others did not find it uncomfortable. However, they indicated that breastfeeding women needed to be given privacy to breastfeed. One male respondent in the IDI said,

*If a woman breastfeeds near me, I will excuse myself for her to do her thing so she can feel comfortable. I will be uncomfortable because I think she is not comfortable with my presence; I wouldn’t be interested in looking at her.* ***Male IDI respondent, Shiashie, East Legon.***

Some women in the FGDs reported no negative feelings when a woman breastfeeds in public.

*Before I learnt about the benefits of breastfeeding, I would feel uncomfortable so I will try to make her feel uncomfortable by looking away. But now knowing all the benefits, it is normal, I won’t feel anything.* ***Female FGD respondent, University Hospital, Legon.***

About 26% of survey respondents disagreed that breastfeeding should be allowed in all public places; 81% also indicated that women should cover up their breast when breastfeeding in public (Table 2). They mentioned that covering the breasts when BIP takes away the feeling of shyness. Majority (70%) also indicated that women should find private places to breastfeed when in public (Table 2).

*You can use a handkerchief or something to cover the breast if you are feeling shy, so that no one sees your breast but then the nipples will be in the baby’s mouth, but if you are not shy, there’s no need to cover it.* ***Female FGD respondent, Shiashie East Legon.***

This perception was contrary to responses from the KIIs where respondents indicated that BIP should be normal practice, and women are expected to breastfeed comfortably without feeling shy anywhere the child demands to be fed. Priority was to be given to the child’s needs.

*I don’t see anything wrong with BIP. It is acceptable and very normal. We have a policy that encourages mothers to initiate and continue breastfeeding on demand. Wherever the baby is demanding for the breastmilk, you have to give it.* ***Male KII respondent, University Hospital, Legon.***

**Table 2:** Women’s perception of breastfeeding in public places

Women’s perception of breastfeeding in public places	% (*n*)
Breastfeeding should be allowed in all public places	
Strongly disagree	14.7 (44)
Disagree	11.3 (34)
Neutral	1.3 (4)
Agree	5.7 (17)
Strongly agree	67 (201)
I should be able to breastfeed anywhere I want	
Strongly disagree	13.7 (41)
Disagree	7.7 (23)
Neutral	1.7 (5)
Agree	5 (15)
Strongly agree	72 (216)
It is acceptable to breastfeed in public	
Strongly disagree	7 (21)
Disagree	5.7 (17)
Neutral	6 (18)
Agree	7 (21)
Strongly agree	74.3 (223)
I feel the need to cover my breast when breastfeeding in public	
No	19 (57)
Yes	81 (243)
It is uncomfortable to watch another woman breastfeed in public	
Strongly disagree	61.0 (183)
Disagree	5.0 (15)
Neutral	2.7 (8)
Agree	9.3 (28)
Strongly agree	22.0 (66)
Women should cover their breasts when breastfeeding in public	
Strongly disagree	8.3 (25)
Disagree	2.7 (8)
Neutral	7.7 (23)
Agree	5.7 (17)
Strongly agree	75.7 (227)
There is a need to find private places when breastfeeding in public	
Strongly disagree	11.7 (35)
Disagree	8.3 (25)
Neutral	9.7 (29)
Agree	5.3 (16)
Strongly agree	65 (195)
It is embarrassing for women to breastfeed in public	
Strongly disagree	65.7 (197)
Disagree	4. 7 (14)
Neutral	2.7 (8)
Agree	10 (30)
Strongly agree	17 (51)
I will breastfeed wherever my child cries for it	
Strongly disagree	18 (54)
Disagree	12.3 (37)
Neutral	1 (3)
Agree	6.7 (20)
Strongly agree	62 (186)
I do not feel comfortable breastfeeding in a public place	
Strongly disagree	46.7 (131)
Disagree	3 (9)
Neutral	1 (3)
Agree	10 (30)
Strongly agree	42.3 (127)
I am comfortable when other women breastfeed their babies near me in public	
Strongly disagree	6 (18)
Disagree	2.7 (8)
Neutral	1.3 (4)
Agree	7.3 (22)
Strongly agree	82.7 (248)
Breastfeeding in public should be against the law	
Strongly disagree	88.3 (265)
Disagree	2 (6)
Neutral	2.7 (8)
Agree	2.7 (8)
Strongly agree	4.3 (13)

### Women’s practice and experience of breastfeeding in public

Majority (93%) of the women in the survey reported ever breastfeeding in public and 81% of them reported feeling the need to cover their breasts when they had to breastfeed in public (Table 2). The FGDs suggest that BIP is practiced by most women to prevent the child from crying and to provide the needed nourishment for the child.

*I don’t like my child to cry in public for people to look at me, so if I see the child acting up, I immediately put the breast in his mouth. I don’t want him to cry, so I breastfeed.* ***Female FGD respondent, Mempeasem***

Fifteen percent of women in this study had observed negative reactions from people when they breastfed in public that made them uncomfortable. The interviews and group discussions also revealed negative reactions of people towards breastfeeding mothers in public places such as at church and the workplace.

*It was Sunday, I was in church and my child was crying. It wasn’t a loud cry but I decided to breastfeed, although I had expressed breastmilk in the bottle. It was a designated place for breastfeeding mothers but the other nursing mothers had bad expressions on their faces. I felt very bad. After that experience, I will not do it again, I will just give the bottle.* ***Female KII1 respondent, University Hospital, Legon.***

About 74% of women indicated that their family or close friends consider the practice of BIP as normal whilst 13% disapproved. About a quarter of women (27.7%) said their partners were uncomfortable with BIP (Table 3).

**Table 3:** Women’s perception of family support for breastfeeding in public places

Family/friends perception of breastfeeding in public	% (*n*)
Has Family/friends breastfeeding a child when respondent was also breastfeeding	
Yes	70 (210)
No	30 (90)
Family or friends support exclusive breastfeeding for six months	
Strongly disagree	22.3 (67)
Disagree	8.3 (25)
Neutral	11.7 (35)
Agree	7 (21)
Strongly agree	50.7 (152)
Family/friends consider breastfeeding in public as normal behavior	
Strongly disagree	7.3 (22)
Disagree	5.7 (17)
Neutral	6 (18)
Agree	7 (21)
Strongly agree	74 (222)
Family/friends encourage me to breastfeed in public	
Strongly disagree	7.7 (23)
Disagree	6.3 (19)
Neutral	8 (24)
Agree	8.7 (26)
Strongly agree	69.3 (208)
Family/friends do not approve of breastfeeding in public	
Strongly Disagree	64 (192)
Disagree	6.3 (19)
Neutral	6.7 (20)
Agree	6 (18)
Strongly Agree	17 (51)
Partner/husband uncomfortable when I breastfeed in public.	
Strongly disagree	64.3 (193)
Disagree	4.3 (13)
Neutral	3.7 (11)
Agree	5 (15)
Strongly agree	22.7 (68)

In the FGDs, women narrated incidents with their families, regarding BIP.

*I delivered at the university hospital, my mother in-law was around me and she wanted me to breastfeed the baby. It was my first time and there were so many people around me. I was feeling shy so I hesitated. She held my breast herself and pulled it out for the baby. I felt bad. Yes. It was my first time and my breasts were very full but she wanted the best for the baby.* ***Female FGD respondent University, Hospital, Legon.***

Participants in the FGDs identified discomfort and impropriety as reasons why BIP is difficult. Women’s education, employment outside of home, and exposure to foreign culture were also reported to negatively affect perception of breastfeeding in public.

*It is actually the foreign culture but I also think most of the women today are working, so they are exposed to the public. Maybe that’s why they don’t feel comfortable exposing their breast in public.* ***Female FGD respondent, Shiashie East Legon.***

Not all women respondents agreed with these views on education and exposure to foreign culture. One woman had this to say:

*I think it’s the individual’s negative perception, Education doesn’t say you should not breastfeed in public, it is the individual’s way of thinking.* ***Female FGD respondent, Shiashie East Legon.***

From the survey, older women (> 25 years) and women with lower than secondary level education showed a positive perception of BIP compared to younger and highly educated women, but this relationship was not statistically significant. From the group discussions it was found that women who perceived BIP positively and see it as acceptable, attributed their perception to awareness of the benefits of breastfeeding at antenatal clinics, family/close friend support of BIP, having many children, and use of breast covers.

*I’m encouraged to breastfeed in public and I find it acceptable because I know breast milk is very good for babies. It protects them, I know all the benefits so even in public I breastfeed.* ***Female FGD respondent, Shiashie East Legon***

Having information on breastfeeding during pregnancy was not associated with positive perception of BIP, although in the FGDs, the women stated that they were encouraged to breastfeed in public at the ANC:

*Although I’m a first time mother, I learnt from the ANC to breastfeed on demand so I learnt to breastfeed in public, although it’s embarrassing. Also in public, you have to cover it if you are feeling shy to make it easier to breastfeed in public.* ***Female FGD respondent, University Hospital, Legon.***

When asked whether their perception about BIP has changed after observing uncomfortable reactions from people, women in the FGD responded:

*No, not at all. I will still do it. As for that, as long as my child cries or I see it is hungry I will give it. If I am shy, I will just cover it. If you are not comfortable you can leave.* ***Female FGD respondent, Shiashie East Legon.***

*I felt very bad, after that experience, I will not do it again, I will just give the bottle.* ***Female KII respondent, University Hospital, Legon.***

As indicated in Table 4, Women who favored BIP, and perceived it as acceptable, were less likely to cover their breast when BIP (OR 0.13, 95% CI 0.03, 0.43). Also, the survey revealed that women who reported strong social support from family and friends were more likely to perceive BIP positively (OR 7.91, 95% CI 4.26, 14.67). Women with strong family support were more likely to practice BIP (OR 3.27, 95% CI 1.31, 8.18). Women were more likely to breastfeed in public if their husbands strongly approved of the practice (OR 3.99, 95% CI 1.5, 10.57).

**Table 4:** Women’s perception and practice of breastfeeding in public places by simple logistic regression analysis

Characteristic	Odds ratio	95% Confidence interval	
Factors associated with women’s positive perception/approval of breastfeeding in public			
Age	1.77	0.71	4.39
Education	0.40	0.09	1.86
Employed	0.89	0.44	1.80
Being married	0.35	0.08	1.56
Parity	1.71	0.68	4.29
Breastfeeding knowledge^a^	1.83	0.64	5.23
Use of breast covers	0.13	0.03	0.43
Negative reactions from people^a^	0.46	0.34	0.89
Family support^a^	7.91	4.26	14.66
Partner’s feeling of comfort with BIP^a^	7.56	4.01	14.27
Factors associated with women’s practice of breastfeeding in public			
Age	0.69	0.23	2.14
Education	0.66	0.27	1.61
Employed	2.08	0.76	5.65
Being married	0.77	0.09	6.09
Parity	2.68	0.32	22.46
Antenatal care	3.44	0.37	32.22
Breastfeeding knowledge	1.99	0.42	9.42
Use of breast covers	0.69	0.19	2.44
Negative reactions from people	1.09	0.31	3.87
Family support^a^	3.27	1.31	8.18
Partner’s feeling of comfort with BIP^a^	3.99	1.50	10.57
Women’s perception score^a^	3.62	1.47	8.91

^a^Statistically significant at p < 0.05. *BIP* Breastfeeding in Public

Controlling for women’s age, education, parity, and employment, family support for BIP was significantly associated with the odds of positive perception (AOR 8.57. 95% CI 4.35, 16.87) but not the practice (AOR 1.86, 95% CI 0.52, 6.62) of breastfeeding in public. Women’s feeling of the need to cover up their breasts when BIP was also significantly associated with a lower odds of positive perception of BIP (AOR 0.12, 95% CI 0.04, 0.40), but not the actual practice (AOR 1.34, 95% CI 0.33, 5.46) (Table 5). Also, women who did not report partner discomfort with BIP were more likely to perceive BIP positively (OR 3.85, 95% CI 1.04, 14.30) (Table 5).

**Table 5:** Factors associated with women’s perception and practice of breastfeeding in public based on multivariate logistic analysis

Characteristic	Adjusted Odds ratio	95% Confidence interval	
Factors associated with positive perception of breastfeeding in public			
Age, y			
< 25	Ref		
25–29	2.13	0.79	5.71
30–34	3.06	1.07	8.79
Education level completed			
No formal education	Ref		
Elementary	0.58	0.11	3.02
Secondary+	0.49	0.10	2.53
Employment status			
Unemployed	Ref.		
Currently employed	0.74	0.34	1.60
Number of live births			
1	Ref		
2–3	0.84	0.44	1.58
> 3	1.52	0.49	4.70
Marital status of respondent			
No relationship	Ref		
In a relationship	0.49	0.10	2.34
Need to cover breast			
No	Ref		
Yes	0.12	0.04	0.40
Family support			
Positive	8.57	4.35	16.87
Factors associated with practice of breastfeeding in public			
Age			
< 25	Ref		
25–29	1.37	0.37	5.07
30–34	1.52	0.41	5.69
Education			
No education	Ref		
Elementary	0.51	0.17	1.51
Number of livebirths			
1	Ref		
2–3	0.74	0.24	2.27
> 3	2.91	0.28	30.07
Family support			
Negative	Ref		
Positive	1.87	0.53	6.62
Need to cover breast			
No	Ref		
Yes	1.34	0.33	5.46
Partner feeling of discomfort			
Yes	Ref		
No	3.85	1.04	14.30

### Breastfeeding in public and bottle feeding

Participants in the FGD and IDI reported that some women avoided BIP because they had alternatives to feeding such as bottle feeding of formula or expressed breastmilk. Urbanization and availability of breast pumps for expressing breast milk were blamed for women not desiring to breastfeed in public.

*Breastfeeding is something that is uncomfortable to do so what I normally do is that when I am going to a public place, I just express into a feeding bottle and keep it in a warmer, I have a warm bag and then when baby is crying, I give the bottle.* ***Female KII respondent, University Hospital, Legon.***

*Formerly, women walked about whiles their babies were breastfeeding but now, advancement in technology has led to feeding with bottles and expressing of breastmilk. So even me now I express my breast milk into a bottle when going to church.* ***Female FGD respondent, Shiashie, East Legon.***

## Discussion

This study was designed to describe women and community perceptions and practice of BIP and its associated factors. The survey results show that BIP is a common practice in Ghana with almost all women having ever breastfed in public. However, women expressed feelings of discomfort, embarrassment, or stigmatization linked with BIP, as reported elsewhere [16]. In-depth interview respondents also mentioned shyness and discomfort as potential barriers to breastfeeding in public. These perceptions about BIP can affect breastfeeding behavior in Ghana since women are increasingly participating in work outside the home. These feeling associated with women’s practice of BIP could negatively affect the choice to initiate or continue breastfeeding among women who may have to be out of their homes for work or other activities while nursing their infants [17].

This first study on BIP in Ghana provides important evidence for further exploring the effect of the external environment on breastfeeding behavior. In a setting where BIP is commonly practiced, the findings also provide a basis for exploring how changes in societal way of life, including working outside of the home, influences breastfeeding practice.

The majority of the survey respondents agreed that their spouses and family or close friends perceived BIP as normal and so encouraged them to do so. Only a small minority did not practice BIP because it made their spouses uncomfortable. A significant association was also found between family or spousal perception of BIP and the women’s own perception and practice of breastfeeding in public. Previously, it has been reported that women were more likely to breastfeed in public if their family/partners supported them to do so [18]. Some women in the FGD mentioned family or close friends as people who helped them to overcome shyness and to breastfeed in public. It is evident that social support from close relations of women is important to help them foster positive behavior toward breastfeeding and consequently improve the duration and quality of breastfeeding practices among these women. In Mitchell-Box and Braun’s study [19], male partners of pregnant women and new mothers, from low income settings reported feeling uncomfortable with breastfeeding in public. As male partners are more likely to influence women’s decision on breastfeeding, there is a need for partner-oriented interventions that encourages support for women and ensure acceptance of breastfeeding in public.

Addressing awareness and fostering positive perceptions among spouses and family of women could be achieved through communication that targets the entire society, including mass media and social media. The ANC which was mentioned by women as a medium by which they were informed about BIP is not usually attended by spouses and relatives. This necessitates the use of other mediums to effectively reach spouses and relatives on their role in supporting women to breastfeed appropriately.

The findings also suggest that women need a safe and secure environment that also offers privacy when breastfeeding outside the home. Elsewhere, it has been reported that women perceived BIP as acceptable in places that provided them some degree of privacy [20]. These findings resonate with a cultural conflict in the Ghanaian setting where young girls are taught from an early age to cover up as critical part of portraying moral decency. Inability to find a private breastfeeding area outside the home could create tensions for breastfeeding women who want to feed their children in a public setting. In the US, feeling embarrassed while breastfeeding in public is considered a formidable barrier to breastfeeding practice [21].

The analysis also showed that women who perceived BIP, negatively, were more likely to cover up during the practice. The covering of the breast is, therefore, a coping mechanism to deal with feeling of discomfort and embarrassment. However, this practice could undermine the eye-to-eye bonding process that occurs between mother and child during breastfeeding. In a hot and humid climate like Ghana, the use of covers during breastfeeding, could make the children feel hot and uncomfortable during breastfeeding [22]. Another coping mechanism for mothers is to avoid breastfeeding in public and instead, to feed the child using a bottle. Bottle feeding is not recommended because it could cause nipple confusion or become a means for microbial contamination. These findings point to the need for public awareness on how to practice BIP and still be able to adhere to breastfeeding recommendations. With the currently low exclusive breastfeeding rates in Ghana and a bottle feeding prevalence of 14% among children aged 1–23 months, and in other low income-countries, it will be crucial to put in measures that eases the discomfort women face with BIP to help reduce the prevalence of bottle feeding. This will encourage women to breastfeed wherever they find themselves, as exclusive breastfeeding requires feeding babies on demand. Preventing women from freely breastfeeding when in public through the negative reactions of people will mean asking them to stay indoors always and hence infringing on their fundamental human right to free movement. The nation can also ensure the rights of women by enforcing regulations such as Ghana’s Labour Law (ACT 651) that permit working women to breastfeed their babies at work.

The participants for the study were selected solely from an urban setting and thus the findings may be applicable to other communities with similar characteristics and thus may not be generalizable to elsewhere in Ghana, in particular rural settings. Further, the study did not collect information on women’s religious or ethnic backgrounds which is a characteristic may be important for explaining women’s perception and practice of breastfeeding in public.

## Conclusions

Breastfeeding in public is viewed by most women as normal and acceptable, and so many of them practice it. Most women expressed a positive perception toward BIP and the majority indicated current or previous practice of breastfeeding in public. However, there were reports of feeling discomfort and embarrassment which can potentially affect desire to breastfeeding in public. Family/partner support was linked with more positive perception and practice of breastfeeding in public. The majority of women felt the need to use breast covers when breastfeeding in public.
